# Predisposing factors, neurological complications and sequels of guillain barre syndrome at discharge: experience in an ICU of a third level hospital

**DOI:** 10.1186/2197-425X-3-S1-A993

**Published:** 2015-10-01

**Authors:** E Portugal Rodríguez, A Berrazueta Sánchez de Vega, R Vara Arlanzón, E Martínez Barrio, M Del Valle Ortiz, JA Fernández Ratero, ME Perea Rodríguez, M Gero Escapa, M Montero Baladía, S Calvo Simal

**Affiliations:** Burgos University Hospital, Burgos, Spain

## Introduction

Guillain Barre Syndrome (GBS) is an autoimmune inflammatory polyneuropathy characterized by flaccid areflexic paralysis, albuminocytologic dissociation in the CSF and demyelination. Clinical weakness is marked by axonal injury not by the demyelination; therefore axonal form has a worse prognosis.

## Objectives

To describe the characteristics of patients admitted with GBS. Analyze its' triggers and severity (APACHE II). To examine subtypes and determine whether patients with axonal form (AF) presented more complications, mortality, sequels and length of stay (LOS) than the patients with demyelinating form (DF). To correlate days of invasive mechanical ventilation (IMV) with complications and sequels at discharge.

## Methods

Descriptive, observational study of all patients with GBS admitted to a general ICU during 13 years. Chi-square and exact Fisher test were used. Variables: sex, age, gravity, stay, subtypes, triggers, complications, and neurological sequels (NS): mild (minor symptoms, enable to run), moderate (able to walk 10 meters unaided), severe (able to walk more than 10 meters with assistance) and very severe (sofa-bed life or need of IMV).

## Results

We analyzed 15 cases of GBS (66.6% males). Mean age 61 (± 19.8). Median APACHEII 11.75 ± 7.1. Mean ICU LOS 63.60 ± 100.2 days and hospital LOS 90.67 ± 116.5. In the electromyographic recording, 50% had DF and 50% had the AF. The 33% had a respiratory trigger, gastrointestinal in 20%, 7% after vaccination and 20% fever of unknown origin. Serologic test were positive in 5 patients. C. jejuni (2), VEB (1), H. pylori (1) and both B.Burdogferi plus C.jenuni (1). Just 13 cases needed IMV with mean duration 39.92 ± 31,52 days. There was extubation failure in 46% of the cases; 80% required tracheostomy. We diagnosed nosocomial infections in 87%, of which 33% was associated with IMV (ventilator associated pneumonia, VAP). The ICU mortality was 20%. No significant relationship (SR) was found between the AF and the presence of more complications, mortality, ICU or hospital LOS. Regarding the NS observed, 16.6 % were mild; 25 % severe, 41.6 % very severe and there was no SR between the worst prognosis and the AF. We found SR between IMV and VAP, with a greater duration of IMV in the group in which VAP is present (p = 0.041). Statistical association exits between longer duration of IMV and severity of sequels at discharge (p = 0.015).

## Conclusions

GBS is a rare disease with low mortality and prolonged hospital and ICU LOS. No SR is evidenced between AF and greater complications, hospital stay, mortality and sequels at discharge. The duration of IMV predicted higher rates of VAP. The severity of sequels at discharge was associated with more days of IMV.
